# Seroprevalence of Anti-SARS-CoV-2 Antibodies in Blood Donors from Nuevo Leon State, Mexico, during 2020: A Retrospective Cross-Sectional Evaluation

**DOI:** 10.3390/v13071225

**Published:** 2021-06-24

**Authors:** Natalia Martinez-Acuña, Diana Minerva Avalos-Nolazco, Diana Raquel Rodriguez-Rodriguez, Cynthia Gabriela Martinez-Liu, Kame Alberto Galan-Huerta, Gerardo Raymundo Padilla-Rivas, Javier Ramos-Jimenez, Sergio Ayala-de-la-Cruz, Eduardo Cienfuegos-Pecina, Erik Alejandro Diaz-Chuc, Rogelio Cazares-Tamez, Amador Flores-Arechiga, Fernando Perez-Chavez, Daniel Arellanos-Soto, Sonia Amelia Lozano-Sepulveda, Elvira Garza-Gonzalez, Consuelo Treviño-Garza, Roberto Montes-de-Oca-Luna, Aurora Beatriz Lee-Gonzalez, Manuel Enrique de-la-O-Cavazos, Ana Maria Rivas-Estilla

**Affiliations:** 1 Department of Biochemistry and Molecular Medicine, School of Medicine, Autonomous University of Nuevo León, Monterrey 64460, Mexico; nmartinez.me0120@uanl.edu.mx (N.M.-A.); dianaxavalos@gmail.com (D.M.A.-N.); raquelrodriguez85@gmail.com (D.R.R.-R.); cmliu@outlook.es (C.G.M.-L.); kame.galanhr@uanl.edu.mx (K.A.G.-H.); gpadillarivas@gmail.com (G.R.P.-R.); d_arellanos_s@yahoo.com (D.A.-S.); lozano_sonia@hotmail.com (S.A.L.-S.); elvira.garzagn@uanl.edu.mx (E.G.-G.); 2 Center of Research and Innovation on Medical Virology, School of Medicine, Autonomous University of Nuevo Leon, Monterrey 64460, Mexico; javramos31@gmail.com; 3 Department of Internal Medicine, Infectious Disease Service, Hospital Universitario “Dr. Jose E. Gonzalez”, Autonomous University of Nuevo León, Monterrey 64460, Mexico; 4 Department of Clinical Pathology and Blood Transfusion Bank, Hospital Universitario “Dr. Jose E. Gonzalez”, Autonomous University of Nuevo León, Monterrey 64460, Mexico; dr.sergioayala.hu@gmail.com (S.A.-d.-l.-C.); eduardo.cienfuegospe@uanl.edu.mx (E.C.-P.); ediaz.me0085@uanl.edu.mx (E.A.D.-C.); dr.cazares07@gmail.com (R.C.-T.); arechiga@outlook.com (A.F.-A.); fernando.perezchv@uanl.edu.mx (F.P.-C.); 5 Department of Pediatrics, Hospital Universitario “Dr. Jose E. Gonzalez”, Autonomous University of Nuevo León, Monterrey 64460, Mexico; cotrevin@hotmail.com (C.T.-G.); delaocavazos@yahoo.com (M.E.d.-l.-O.-C.); 6 Secretariat of Health of Nuevo León State, Monterrey 64460, Mexico; roberto.montesdeocaln@uanl.edu.mx (R.M.-d.-O.-L.); beatriz.lee@saludnl.gob.mx (A.B.L.-G.); 7 Department of Histology, School of Medicine, Hospital Universitario “Dr. Jose E. Gonzalez”, Autonomous University of Nuevo León, Monterrey 64460, Mexico; 8 Transfusion Center, CETS, Secretariat of Health of Nuevo Leon State, Monterrey 64460, Mexico

**Keywords:** blood donors, COVID-19, anti-SARS-CoV-2 IgG, seroprevalence, Mexico

## Abstract

The progression and distribution of the SARS-CoV-2 pandemic are continuously changing over time and can be traced by blood donors’ serological survey. Here, we investigated the seroprevalence of anti-SARS-CoV-2 antibodies in blood donors in Nuevo Leon, Mexico during 2020 as a strategy for the rapid evaluation of the spread of SARS-CoV-2 and asymptomatic case detection. We collected residual plasma samples from blood donors who attended two regional donation centers from January to December of 2020 to identify changes in anti-SARS-CoV-2 IgG prevalence. Plasma samples were analyzed on the Abbott Architect instrument using the commercial Abbott SARS-CoV-2 IgG chemiluminescent assay. We found a total of 99 reactive samples from 2068 analyzed plasma samples, resulting in a raw prevalence of 4.87%. Donors aged 18–49 years were more likely to be seropositive compared to those aged >50 years (*p* < 0.001). Weekly seroprevalence increased from 1.8% during the early pandemic stage to 27.59% by the end of the year. Prevalence was 1.46-fold higher in females compared to males. Case geographical mapping showed that Monterrey city recorded the majority of SARS-CoV-2 cases. These results show that there is a growing trend of seroprevalence over time associated with asymptomatic infection that is unnoticed under the current epidemiological surveillance protocols.

## 1. Introduction

**Figure 1:**
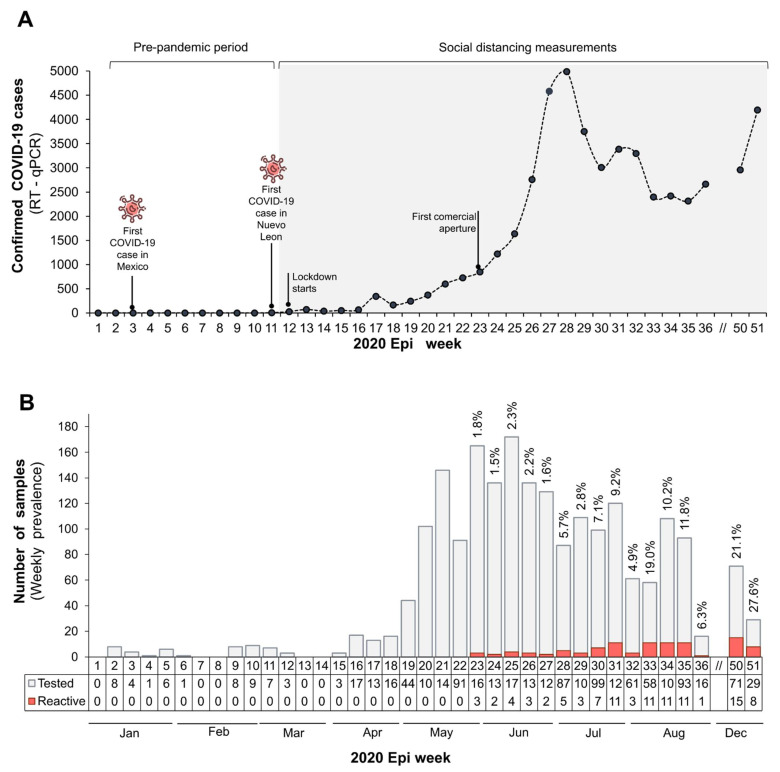
Dynamics of asymptomatic SARS-CoV-2 cases detected by anti-SARS-CoV-2 IgG analysis in blood donors during 2020 in Nuevo Leon, Mexico. (**A**) Curve of RT-qPCR-confirmed new COVID-19 cases per epi week recorded in Nuevo Leon. The gray shading indicates the pandemic period. Significant pandemic events for the Nuevo Leon area are indicated. (**B**) Prevalence of anti-SARS-CoV-2 IgG per epi week was estimated by dividing the number of positive cases (red bars) by the number of tested samples (gray bars) per week. S/C index ≥ 1.4 was considered as a positive result. The respective seroprevalence of SARS-CoV-2 IgG is shown on the top of each bar.

Severe acute respiratory syndrome coronavirus 2 (SARS-CoV-2), producing coronavirus disease 2019 (COVID-19), has spread worldwide, becoming a significant public health problem. At this time (22 May 2021), it has caused over 166 million cases worldwide and at least 3.4 million deaths [1]. Mexico is one of the most affected countries in Latin America, with 2,392,744 confirmed infections and 221,256 deaths [1]. In the state of Nuevo Leon (Northeast Mexico), the pandemic has caused 177,164 total cases and 10,777 deaths [2]. Nuevo Leon, located at the northern frontier that borders the US state of Texas, hosts one of the three biggest cities in Mexico. Due to its economic activity, which is based on manufacturing, commercial business, and education, 96% of its 5.6 million inhabitants are concentrated around the urban area of Monterrey [3]. Due to the high population density and the continuous population displacement in Nuevo Leon, as soon as the virus was detected in the region (March 2020), the state closed schools and restricted commercial activities and social meetings. After a series of governmental decisions, Mexico planned a gradual re-opening of its economy in early June 2020, causing an expected increase in the number of COVID-19 cases (Figure 1). The clinical presentation of COVID-19 in patients includes fever, dry cough, and fatigue as the most common symptoms that may appear 3–14 days after infection. Several reports have suggested that SARS-CoV-2 infections can be asymptomatic. It is reported that the asymptomatic infection incidence can range from 1.2 to 12.9% [4,5] in large populations. However, other studies reported a much higher proportion reaching up to 87.9% [6,7]. Reports have demonstrated that viral loads are very similar in symptomatic and asymptomatic groups, making the latter capable of spreading SARS-CoV-2 despite the absence of clinical manifestations [8,9]. Mexico has not established a screening protocol to identify asymptomatic cases, but it is necessary to take both symptomatic and asymptomatic infected subjects into account in order to obtain more detailed epidemiologic data and recalculate the prevalence and fatality rates of the COVID-19 pandemic.

Estimating the seroprevalence of anti-SARS-CoV-2 antibodies (Abs) in blood donors is a powerful and cost-effective strategy to monitor the population’s exposure and detect asymptomatic SARS-CoV-2 cases. After infection occurs, specific Abs against SARS-CoV-2 appear between four and five days (IgM, immunoglobulin M), and most of the patients seroconvert (IgG, immunoglobulin G) within the first three weeks [10,11,12]. Robust titers of specific Abs can be detected up to five months post-SARS-CoV-2 infection [13].

Early studies focusing on blood donors to identify SARS-CoV-2-infected persons were conducted in different regions [14,15]. The seroprevalence of anti-SARS-CoV-2 determined using this approach varies from 0.1% (San Francisco Bay Area) [16] to 5.6% (Kenya) but can reach values of up to 9% when they are adjusted for a specific geographical region [17].

To date, limited data have documented the prevalence of SARS-CoV-2 antibodies in the general population and asymptomatic outpatients in Mexico [18,19]. In this study, we conducted a retrospective cross-sectional seroprevalence survey using residual plasma samples to identify SARS-CoV-2-specific IgG antibodies among blood donors who attended two regional transfusion centers during 2020. The identification of asymptomatic cases of SARS-CoV-2 allowed us to add information to track the progression of the pandemic in our country.

## 2. Materials and Methods

### 2.1. Study Design and Subjects

We performed a retrospective cross-sectional anti-SARS-CoV-2 IgG serological survey study on residual plasma samples from blood donors who attended the Blood Bank Center at “Dr. Jose E. Gonzalez” University Hospital and the Blood Bank of the Transfusion Center of the Secretariat of Health of Nuevo Leon in Northeast Mexico during 2020. Each sample was identified with a numeric code for privacy protection. Sociodemographic data, hematic biometry values, and somatometric parameters were taken from the donor management database of each blood bank. All blood donors were aged 18–65 years and met the requirements of the Mexican Official Norm NOM-253-SSA1-2012.

During the donation protocol, all donors underwent a medical interview and were asked whether they had experienced any symptoms that suggested respiratory illness or any other infectious disease in the last 30 days. Donors with risk factors for blood-transmitted diseases, recent disease, or who were deemed unhealthy during the physical examination were excluded. Once the donation was approved, blood was fractionated and the fresh plasma was kept at −30 °C until use.

### 2.2. Serological Analysis

For antibody testing, we performed a chemiluminescent microparticle immunoassay (CMIA) for the qualitative detection of IgG antibodies against SARS-CoV-2 nucleoprotein using an anti-SARS-CoV-2 IgG kit (06R8620, from Abbott Laboratories, Abbott Park, IL, USA).

Plasma samples were run in an ARCHITECT system (from Abbott Diagnostics, Abbott Park, IL, USA following the manufacturer’s instructions. This laboratory test was only used to determine past infection events. In this study, an index sample/calibrator (S/C) threshold of 1.4 or greater was taken as a positive result. FDA analysis reported sensitivity and specificity values of 90% and 100%, respectively (data available in https://www.accessdata.fda.gov/cdrh_docs/presentations/maf/maf3305-a001.pdf, accessed: 22 May 2021) and no significant cross-reactivity has been observed [20,21].

Serologic routine tests against *Treponema pallidum* (VDRL), hepatitis C virus (HCV), human immunodeficiency virus type 1 (HIV-1), hepatitis B virus (HBV), and *Trypanosoma cruzi* (Chagas disease) antibodies were run in an ARCHITECT i2000 SR analyzer (Abbott Diagnostics, Chicago, IL, USA). The presence of antibodies against *Brucella* was determined by the Rose Bengal Test (Licon, Mexico City, Mexico), a rapid slide-type agglutination assay.

### 2.3. Data Analysis

We tabulated the data in a Microsoft Excel^®^ spreadsheet with donors’ demographic characteristics (sex, age, blood type, and education level) and test results, reported by numeric code for privacy. The date of the donation was used as a reference to plot cases per epidemiological week (epi week), and the address was used for geographical mapping.

The raw SARS-CoV-2 prevalence in blood donors and the adjusted prevalence according to the sensitivity and specificity of the assay were calculated [22]. Prevalence was estimated as total prevalence (number of IgG-positive samples/total tested samples), weekly prevalence (number of IgG-positive samples/number of analyzed samples per epi week), and by donor demographic characteristics (IgG-positive samples/number of analyzed samples per group). According to the data type, we performed a χ^2^ test for categorical variables expressed as frequencies or percentages for descriptive statistics, using GraphPad Prism 9.1 software. To evaluate the association between SARS-CoV-2 infection and potential risk factors, we used logistic regression models and odds ratios (ORs). Statistical tests at a 5% significance level were adopted for relating the prevalence of anti-IgG antibodies to SARS-CoV-2 to donors’ characteristics (sex, age group, blood type, and education level). Statistical analysis was performed using SPSS software.

The donors’ home addresses were used to map the cases to the geographical distribution across Nuevo Leon using ArcGIS v10.2.2 (ESRI, Redlands, CA, USA). Official records of COVID-19 cases, confirmed by RT-qPCR (quantitative reverse-transcription PCR), were obtained from epidemiological reports available from https://www.nl.gob.mx/publicaciones/casos-de-covid-19-en-nuevo-leon, last accessed 22 may 2021). Cases detected by RT-qPCR represent mainly symptomatic individuals who attended COVID-19 statal attention centers.

## 3. Results

### 3.1. Study Population

A total of 2068 plasma samples from blood donors were obtained from 1 January to 15 December 2020. Males contributed 73.83% (1527/2068) of the plasma specimens, and 26.1% (541/2068) were from females. The median age of subjects studied was 34 years (IQR 16 years). The samples were grouped per epi week and divided into two observational periods: the pre-pandemic period (epi week 1 to 10) and the pandemic phase (epi week 11 to 52) (Figure 1A). Based on these criteria, 37 plasma samples were classified as pre-pandemic and 2031 as pandemic. This last subset was used for data analysis and seroprevalence estimation.

### 3.2. Detection of Anti-SARS-CoV-2 IgG in Plasma Derived from Blood Donors

All plasma samples from the pre-pandemic period tested negative for anti-SARS-CoV-2 IgG. However, we detected 99 reactive plasma samples among the pandemic group, leading us to estimate a raw prevalence of 4.87% and 5.3% after adjusting prevalence for test specificity and sensitivity. Of the 99 positive cases, 65 were males (65.65%) and 34 were females (34.45%).

**Figure 2:**
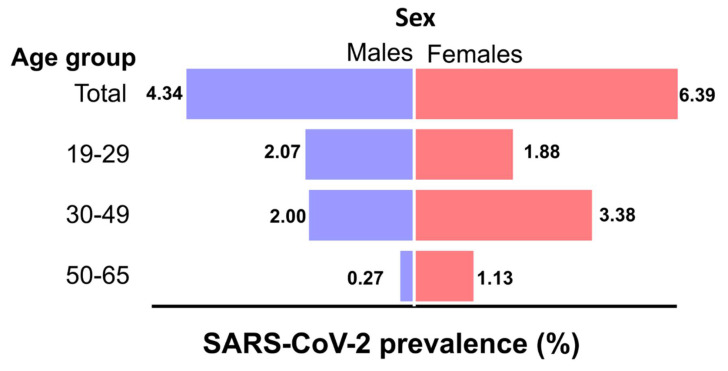
Sex and age distribution of SARS-CoV-2 IgG (+) samples. *n* = 99/2031.

The main characteristics of the pandemic samples group and its specific prevalence values calculated for gender, age group, blood type, and education level are shown in Table S1. Despite the male-to-female ratio of 2.80:1, we observed that the incidence was lower in males (4.34%) than females (6.36%) (*p* = 0.0436) (Figure 2, Table S2). Seroprevalence was ~1.5-fold higher among female donors compared to males. Persons with a higher education level were more exposed to SARS-CoV-2; prevalence in this group was 9.29% (*p* < 0.0001). While blood of types B (5.04%) and AB (6.25%) was more likely to test positive, there was no significant difference from the other groups. Based on the clinical history and medical interviews, reactive plasma can be considered as evidence of asymptomatic SARS-CoV-2 infection.

Temporal distribution analyses showed that the first IgG-positive detection dated from 31 May, which was 12 weeks after the first confirmed COVID-19 case in Nuevo Leon (Figure 1B). We observed an increasing trend in weekly seroprevalence from 1.8% in May (epi week 23) to 19% in August (epi week 33), to 27.59% by the end of the year (epi week 51). Asymptomatic case dynamics tended to be similar to the curve of weekly RT-qPCR-confirmed cases reported by health instances, but in a delayed manner (Figure 1A).

**Figure 3:**
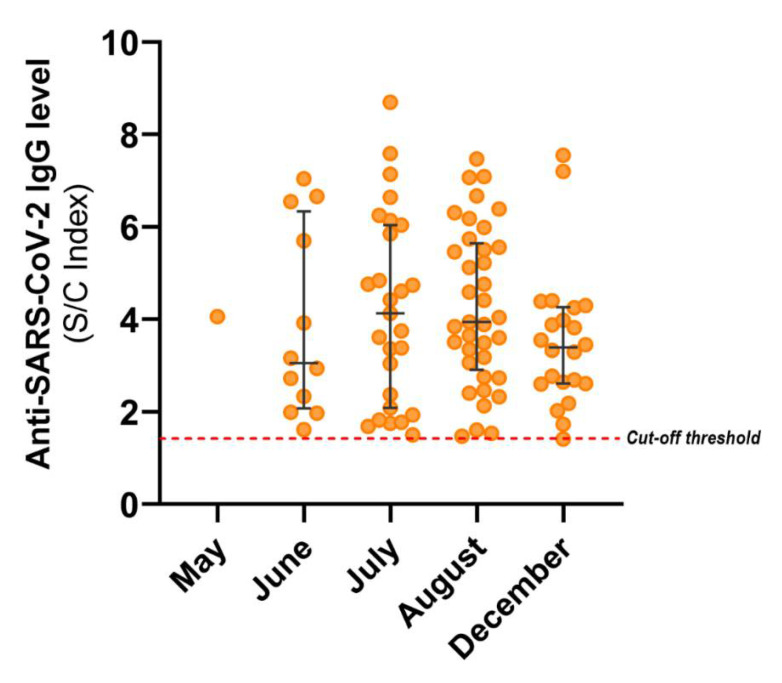
Anti-SARS-CoV-2 IgG titers across the collection period. S/C index values for the 99 reactive samples are graphed per month. Median and IQR are shown. Variation of antibody titers was not significant.

Comparison of antibody titers showed no significant variation between antibody titers among reactive samples and different dates of collection (Figure 3).

### 3.3. Geographical Distribution of Observed Asymptomatic SARS-CoV-2 Infections

**Figure 4:**
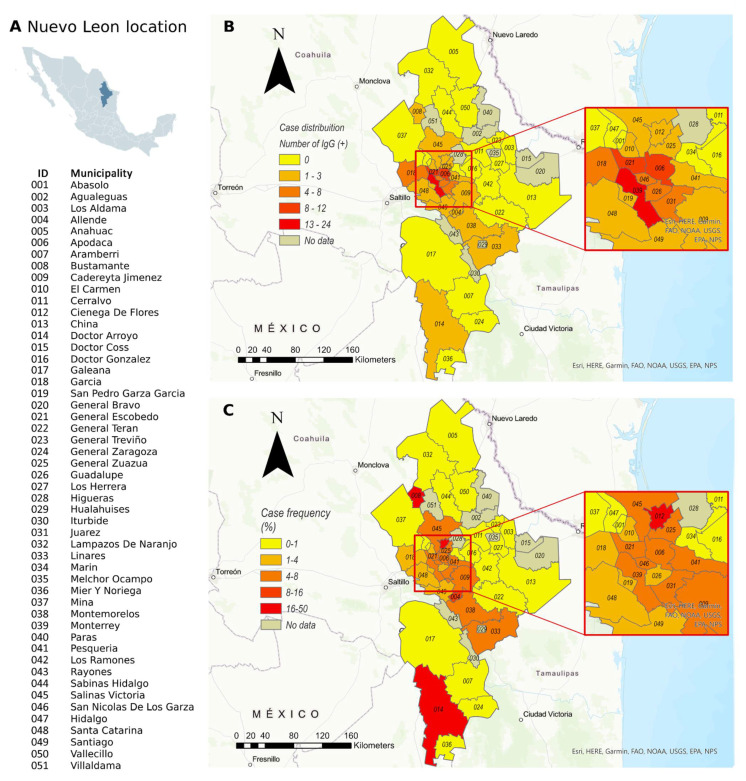
Geographical distribution of asymptomatic SARS-CoV-2 infection events determined by anti-SARS-CoV-2 IgG detection in the Nuevo Leon area. (**A**) Location of Nuevo Leon State in Mexico and municipalities. (**B**) Absolute number of cases per municipality detected during our study. (**C**) Proportion of cases to samples per municipality.

Plasma samples were collected in 41/51 municipalities of Nuevo Leon (Table S3). The 99 SARS-CoV-2 IgG-positive cases observed were distributed across 21 of Nuevo Leon’s municipalities, with Monterrey, Apodaca, and Escobedo contributing almost half (46.46%) of the observed cases (Figure 4B).

The specific prevalence varied depending on the geographical zone. Inside the metropolitan area, specific region prevalence varied noticeably from 1.1% (1/92) in Santa Catarina to 6.5% (12/184) in Apodaca, but in rural areas, rates were higher and reached values over 30% (Figure 4C).

## 4. Discussion

It has been estimated that 15% to 46% of SARS-CoV-2 infections are asymptomatic [23], but these rates can vary widely according to a population’s variations of exposure over time, the epidemiological management of the pandemic, testing methods, and other still-unknown factors that affect different regions in the world.

Despite the expected fluctuation of the seroprevalence throughout the year, in this study we found a clear increase in asymptomatic SARS-CoV-2 cases in the studied population. During 2020, the prevalence increased from 1.8% (epi week 23) to 27.59% (epi week 51) with an average of 19% (epi week 33) (Figure 1B). The increasing seroprevalence observed among the Nuevo Leon population indicates an important elevation in SARS-CoV-2 exposure and contagion in our region. Due to the tested group consisting mainly of healthy subjects, IgG-positive cases can be classified as asymptomatic SARS-CoV-2 cases.

The results showed that asymptomatic SARS-CoV-2 infections were more frequent in females, contrary to males who tend to be symptomatic with higher morbidity and mortality [24]. This observation can be attributed to the fact that the innate antiviral immune response is higher in women [25].

Regarding the overall seroprevalence data reported here (4.87% adjusted to 5.3%), we found a higher prevalence than previously reported in blood donors in Wuhan, China with 2.29% [26], the Netherlands with 2.7% [15], and Denmark with 1.7% [14] at the beginning of the pandemic. In a similar study, the American Red Cross in the United States tested all blood donations from June to August 2020 for anti-SARS-CoV-2 IgG antibodies and found that 1.82% (17,336/953,926) of donations were positive [27]. We also found, as in other regions, that anti-SARS-CoV-2 seroprevalence in blood donors initially grew slowly and then rapidly, but varied by region, as demonstrated by a report from the United Kingdom [28]. Similarly, analysis of the distribution of samples showed that most of these samples were from urban areas and regions with a high population density.

Until now, there have been few studies on the prevalence of SARS-CoV-2 in Mexico. One of them was focused on a specific area (Guadalupe, a municipality in Nuevo Leon) [19], while the other was conducted in another state (Veracruz State in Southeast Mexico) [18]. Both involved cross-sectional analyses during July 2020. Prevalence among asymptomatic subjects from Guadalupe, Nuevo Leon (4.15%, 114/2741) was within the reported rates (1.6–9.2%) for the same period. In contrast, values observed in Nuevo Leon differed greatly from those observed in Veracruz, where 21% of healthy subjects tested positive, showing that the pandemic evolved differently in each city, and control measures needed to be adapted accordingly.

Despite the low risk of SARS-CoV-2 in blood transfusions [29], the prevalence of this new viral agent was higher than other infectious diseases commonly analyzed in blood donors (Figure S1). SARS-CoV-2 increased over time while infections caused by *Brucella spp,* HCV, HIV-1, *Trypanosoma cruzi*, and *Treponema pallidum* remained similar to previous reports in the region [30,31].

Titers of SARS-CoV-2-specific antibodies can remain elevated and stable for up to five months [13]. In this study, samples were collected continuously during the five months after diagnosis of the first confirmed case by RT-qPCR in Nuevo Leon. For this reason, results are unlikely to be affected by a drop in SARS-CoV-2 antibody levels. In addition, we performed a second collection in December to determine if antibody level distribution had changed in a significant manner since August; antibody levels among positive cases remained similar (Figure 3). However, future seroprevalence studies need to be done to assess the lifespan of the antibodies.

As for the limitations of our study, first, we note that the estimated seroprevalence may not reflect the true underlying proportion of those exposed to SARS-CoV-2 in our country because blood donors are not representative of the overall population; second, we did not perform virus neutralization assays, therefore the neutralizing activity of the detected IgG antibodies is unknown; third, a rapid decline in antibody titers and pro-inflammatory cytokines may be a common feature of non-severe SARS-CoV-2 infection, suggesting that asymptomatic individuals may have a weaker immune response to SARS-CoV-2 infection in contrast to symptomatic subjects [32]; fourth, at the time of this work, due to government regulations in Mexico, SARS-CoV-2 serological tests available for diagnostics were limited to IgG analysis. For this reason, we only detected infections with at least two or three weeks of evolution; more recent infections may have gone unnoticed, meaning prevalence could be higher.

Finally, the results of this study allow us to highlight two important points: there is a growing trend of seroprevalence over time, parallel to the constantly increasing epidemic curve in our region, and the higher prevalence of positive plasma found in female subjects under 49 years of age is associated with asymptomatic infection in these donors. This work demonstrated that screening blood donors for SARS-CoV-2 antibodies strengthens existing evidence that this group can be used as a sentinel population to track the progression of the COVID-19 pandemic.

## Data Availability

All primary data presented in this study are available from the corresponding author upon reasonable request. Primary data exist for all figures.

## Supplementary Materials

The following are available online at https://www.mdpi.com/article/10.3390/v13071225/s1, Figure S1: Comparison of prevalence of SARS-CoV-2 IgG antibodies observed against other blood-borne infectious diseases during the same period, Table S1:Blood donor’s characteristics and crude prevalence of antibodies to SARS-CoV-2 in the collected samples from Nuevo Leon Area, Mexico, during the pandemic period (epi week 11 to 51 of 2020),Table S2: Variable association to SARS-CoV-2 risk of infection, Table S3: Geographical distribution of tested samples across Nuevo Leon region and COVID-19 positivity ratio observed.
