# Supplementary material for: Seroprevalence of Anti-SARS-CoV-2 Antibodies in Blood Donors from Nuevo Leon State, Mexico, during 2020: A Retrospective Cross-Sectional Evaluation
Source: Viruses. 2021 Jun 24;13(7):1225. doi: 10.3390/v13071225 (PMC8310175; doi:10.3390/v13071225)
Supplement: Supplementary file 1 [file viruses-13-01225-s001.zip › viruses-1206453suppforXMLFinal.pdf]

Supplementary Materials

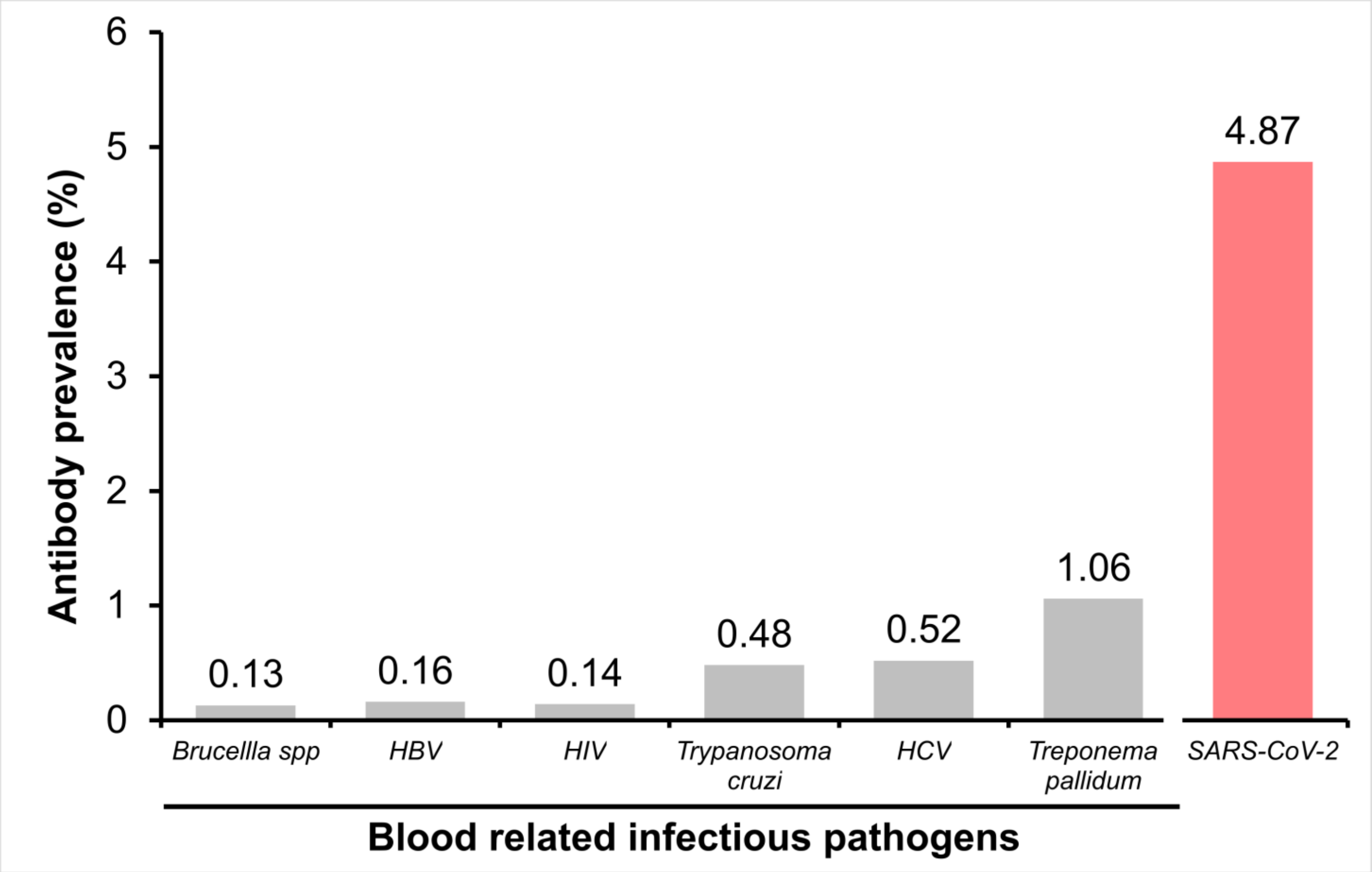

Figure S1. Comparison of prevalence of SARS-CoV-2 IgG antibodies observed against other blood-borne infectious diseases during the same period.

Table S1. Blood donor´s characteristics and crude prevalence of antibodies to SARS-CoV-2 in the collected samples from Nuevo Leon Area, Mexico, during the pandemic period (epi week 11 to 51 of 2020).

Variable	Group	Collected samples (n=2031)	Seropositive cases	Case distribution per group (%)	Prevalence per group	*p*-value[a]
**Pandemic Period**	**Total**	**2031**	**(n = 99)**	**-**	**4.87%**	
**Sex**	**M**	1497	65	65.65%	4.34%	0.0436
	**F**	534	34	34.46%	6.36%	
**Age group**	**18-29**	742	41	41.41%	5.53%	0.514
	**30-49**	1098	48	48.48%	4.37%	
	**50-65**	191	10	10.10%	5.24%	
**ABO blood type**	**A**	501	28	28.28%	5.59%	0.8306
	**B**	139	7	7.07%	5.04%	
	**AB**	16	1	1.01%	6.25%	
	**O**	1375	63	63.64%	4.58%	
**RH Blood type**	**+**	1946	94	94.95%	4.83%	0.6593
	**-**	85	5	5.05%	5.88%	
**Educationa l level**	**Basic**	962	19	19.19%	1.98%	<0.0001
	**Middle**	617	38	38.38%	6.16%	
	**Higher**	452	42	42.42%	9.29%	

Table S2. Variable association to SARS-CoV-2 risk of infection

	OR	95% C.I.		*P**
		Lower	Upper	
**Sex**				
Female	1			
Male	0.679	0.436	1.058	0.087
**Age**				
19-29	1			
30-49	0.823	0.517	1.310	0.411
50-65	0.924	0.429	1.994	0.841
**ABO Blood Type**				
A	1			
B	1.038	0.437	2.463	0.932
O	0.862	0.533	1.395	0.546
AB	1.183	0.148	9.458	0.874
**RH**				
Negative	1			
Positive	0.759	0.298	1.936	0.564
**Educational Level**				
Basic	1			
Medium	1.132	0.700	1.832	0.613
High	0.818	0.457	1.463	0.498

Table S3. Geographical distribution of tested samples across Nuevo Leon region and COVID-19 positivity ratio observed.Nd= No data.

Municipality	Samples	IgG(+)	%
**Abasolo**	3	0	0.0%
**Agualeguas**	0	Nd	Nd
**Los Aldama**	3	0	0.0%
**Allende**	14	2	14.3%
**Anahuac**	6	0	0.0%
**Apodaca**	185	12	6.5%
**Aramberri**	5	0	0.0%
**Bustamante**	6	1	16.7%
**Cadereyta Jimenez**	48	3	6.3%
**El Carmen**	32	1	3.1%
**Cerralvo**	3	0	0.0%
**China**	4	0	0.0%
**Cienega De Flores**	6	2	33.3%
**Doctor Arroyo**	2	1	50.0%
**Doctor Coss**	0	Nd	Nd
**Doctor Gonzalez**	5	0	0.0%
**Galeana**	10	0	0.0%
**Garcia**	158	6	3.8%
**San Pedro Garza Garcia**	37	1	2.7%
**General Bravo**	0	Nd	Nd
**General Escobedo**	193	10	5.2%
**General Teran**	1	0	0.0%
**General Treviño**	1	0	0.0%
**General Zaragoza**	1	0	0.0%
**General Zuazua**	19	1	5.3%
**Guadalupe**	223	8	3.6%
**Los Herrera**	2	0	0.0%
**Higueras**	0	Nd	Nd
**Hualahuises**	0	Nd	Nd
**Iturbide**	0	Nd	Nd
**Juarez**	164	8	4.9%
**Lampazos De Naranjo**	1	0	0.0%
**Linares**	19	1	5.3%
**Marin**	2	0	0.0%
**Melchor Ocampo**	0	Nd	Nd
**Mier Y Noriega**	4	0	0.0%
**Mina**	4	0	0.0%
**Montemorelos**	20	1	5.0%

**Monterrey**	499	24	4.8%
**Paras**	0	Nd	Nd
**Pesqueria**	25	2	8.0%
**Los Ramones**	3	0	0.0%
**Rayones**	0	Nd	Nd
**Sabinas Hidalgo**	8	0	0.0%
**Salinas Victoria**	23	1	4.3%
**San Nicolas De Los Garza**	115	6	5.2%
**Hidalgo**	8	0	0.0%
**Santa Catarina**	92	1	1.1%
**Santiago**	28	1	3.6%
**Vallecillo**	1	0	0.0%
**Villaldama**	0	Nd	Nd
**No Municipality**	2	1	
**Other States/**	83	5	
